# Peritoneal tuberculosis mimicking advanced ovarian carcinoma: an important differential diagnosis to consider

**DOI:** 10.1186/1756-0500-6-88

**Published:** 2013-03-09

**Authors:** Maria A Gosein, Dylan Narinesingh, Gordon V Narayansingh, Nazreen A Bhim, Pearse A Sylvester

**Affiliations:** 1 Department of Radiology, San Fernando General Hospital, San Fernando, Trinidad; 2 Department of Oncology, San Fernando General Hospital, San Fernando, Trinidad; 3 Department of Medicine, University of the West Indies, St. Augustine, Trinidad

**Keywords:** Abdominal disseminated tuberculosis, Mimic ovarian carcinoma, CA125, Ascites, Adnexal mass

## Abstract

**Background:**

Female patients who present with ascites, adnexal masses and elevated CA125 levels are typically presumed to have advanced ovarian carcinoma. This can lead to radical surgery with its associated morbidity. An important differential diagnosis to consider is tuberculous peritonitis which can present in a similar manner and responds well to medical treatment.

**Case presentation:**

A 44 year old female presented with abdominal distension, weight loss and low grade fever. Her CA125 level was 909 U/ml. Imaging studies revealed an adnexal lesion and ascites. The lungs appeared normal and a Mantoux test was negative. Ovarian malignancy was highly suspected. Cytology of ascites was negative for malignant cells. The patient subsequently developed a large pleural effusion which was drained and negative for malignant cells and acid fast bacilli. Repeat imaging revealed a ‘tree in bud’ appearance of the lung parenchyma and dense ascites. Histology from diagnostic laparotomy revealed caseating granulomas with epithelioid cells and Langhan’s type giant cells. The patient responded well to antituberculosis therapy with normalization of CA125 levels, confirming the diagnosis of peritoneal tuberculosis.

**Conclusion:**

CA125 levels lack specificity, with elevated levels encountered in many benign and malignant conditions, including tuberculosis. There are a few discriminating features that suggest a diagnosis of tuberculous peritonitis rather than ovarian carcinoma. Apart from chest findings which may not always be present, smooth peritoneal thickening and a dirty omentum on CT favours a diagnosis of peritoneal tuberculosis compared with nodular thickening of the peritoneum and omentum in peritoneal carcinomatosis. PCR and ADA testing of ascitic fluid can also be helpful. When these tests are negative or unavailable then diagnostic laparoscopy or laparotomy should be performed with the aid of frozen section to avoid unnecessary radical surgery in cases of peritoneal tuberculosis.

## Background

Ovarian carcinoma is typically assumed in a female patient with ascites, adnexal masses and elevated CA125 levels. As extensive debulking is often necessary for ovarian carcinoma, this diagnosis usually leads to radical surgery including the removal of the uterus and ovaries. Disseminated tuberculosis however can present in a similar manner but often responds to medical management. Factors such as the HIV epidemic and increased migration have caused a resurgence of tuberculosis. This differential diagnosis therefore must be considered, particularly in low income and immigrant populations where the incidence is higher [1]. There have been a number of similar cases reported in the literature [2-4] however more than two thirds of patients are diagnosed incidentally after laparotomy [5], where unnecessary radical surgery was performed [6,7]. We present a case of a premenopausal woman diagnosed with peritoneal tuberculosis, initially thought to have ovarian carcinoma. Suspicion for a benign alternate diagnosis was raised when her CA125 level fell drastically on its own, without any treatment, which to our knowledge has not been reported in malignant conditions. We discuss the radiological and clinical findings of peritoneal tuberculosis and discriminating features that aid in the differentiation from ovarian carcinoma.

## Case presentation

**Figure 1:**
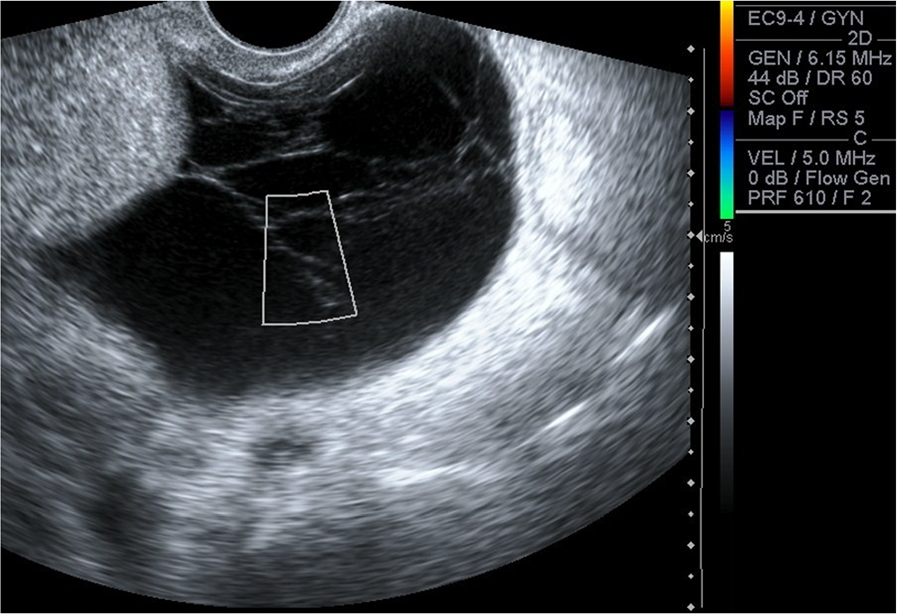
**Transvaginal Ultrasound.** Multilocular cystic left adnexal lesion measuring 7.6 cm x 7.0 cm x 6.1 cm. Septae are thin (<3 mm). No demonstrable solid or vascular component.

**Figure 2:**
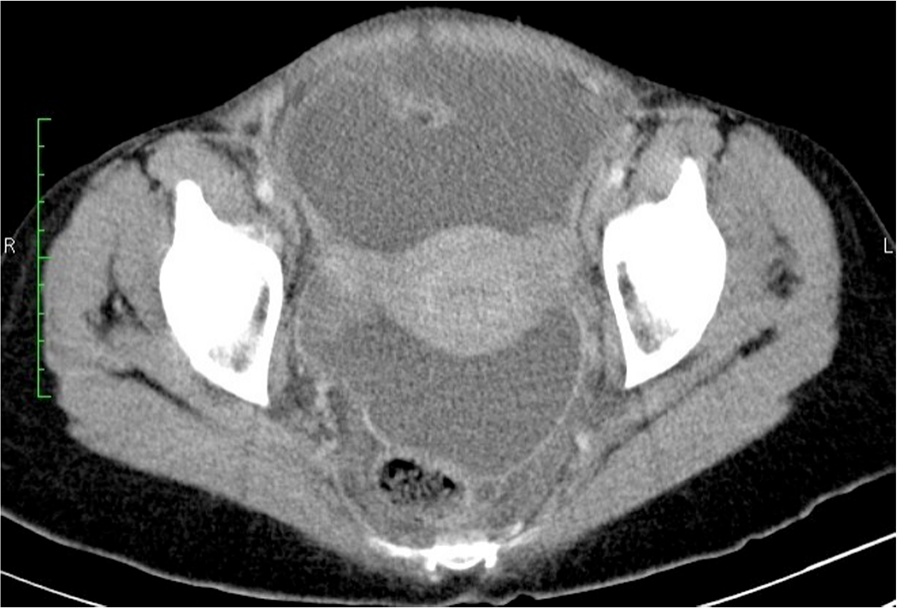
**Contrast Enhanced CT of the pelvis.** Moderate ascites with smooth thickening and strong enhancement of the peritoneum.

A 44 year old afro-Trinidadian female presented with a two week history of abdominal distension, weight loss, decreased appetite and low grade fever. She had a normal white cell count, a haemoglobin of 9.02 g/dL and a CA125 level of 909 U/ml. Ultrasound scan showed moderate ascites with a septated cystic left adnexal mass (Figure 1). CT scan showed smooth peritoneal thickening (Figure 2). There was no lymphadenopathy or peritoneal or hepatic deposits. Chest CT showed no lung parenchyma lesions. An ascitic tap was negative for malignant cells and a mantoux test was negative. Primary ovarian malignancy was suspected and the patient was referred to the gyne-oncology clinic. One week later her symptoms improved and the CA125 level fell to 303 U/ml. Due to this unexpected result, alternate diagnoses were considered. The patient declined to have a diagnostic laparotomy at this time as her symptoms had resolved. She agreed however to be monitored in the clinic but was counselled on the need for definitive diagnosis due to the possibility of ovarian carcinoma.

**Figure 3:**
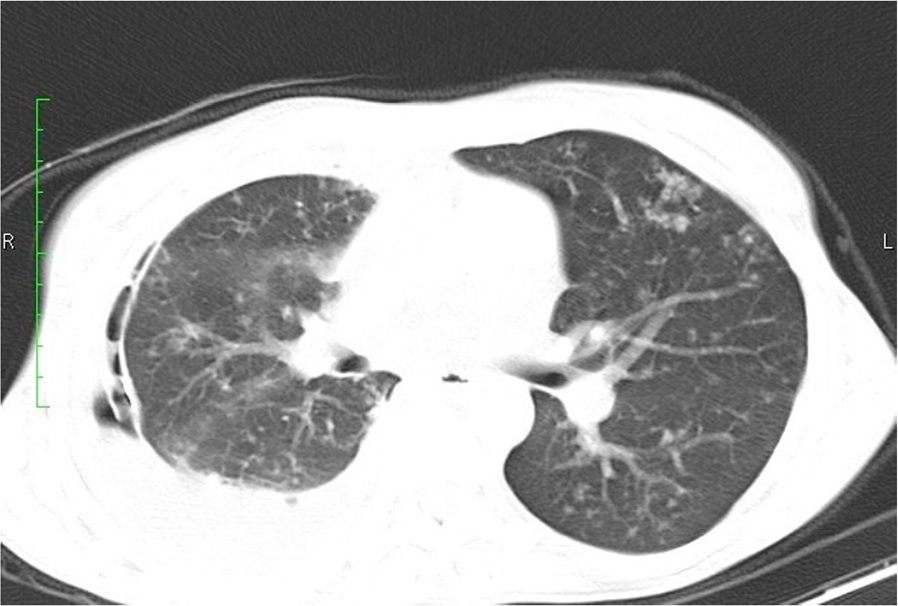
**CT Chest; pulmonary window.** Post chest tube insertion for right pleural effusion. Multiple subcentimeter lung nodules are seen with a ‘tree in bud’ appearance representing active endobronchial spread of disease.

**Figure 4:**
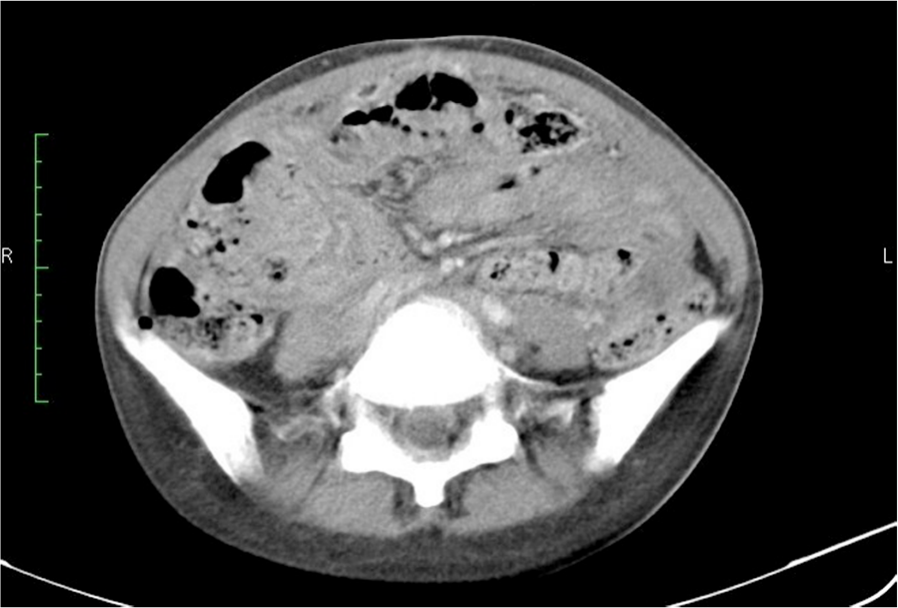
**Contrast Enhanced CT of the abdomen.** Matted bowel loops with mesenteric stranding and dense ascites (Hounsfield unit greater than fluid density).

A few months later she developed dyspnoea due to a large right pleural effusion. A chest tube was inserted and the pleural fluid was negative for malignant cells and acid-fast bacilli (AFB). Repeat CT showed multiple subcentimeter nodules as well as a ‘tree in bud’ appearance throughout the lung parenchyma (Figure 3). The ascitic volume decreased but became denser. Bowel loops appeared matted with mesenteric stranding (Figure 4). In view of the radiological findings tuberculosis was strongly suspected in spite of the negative skin and bacteriologic tests.

The patient then agreed to diagnostic laparotomy (laparoscopy services were not available).

**Figure 5:**
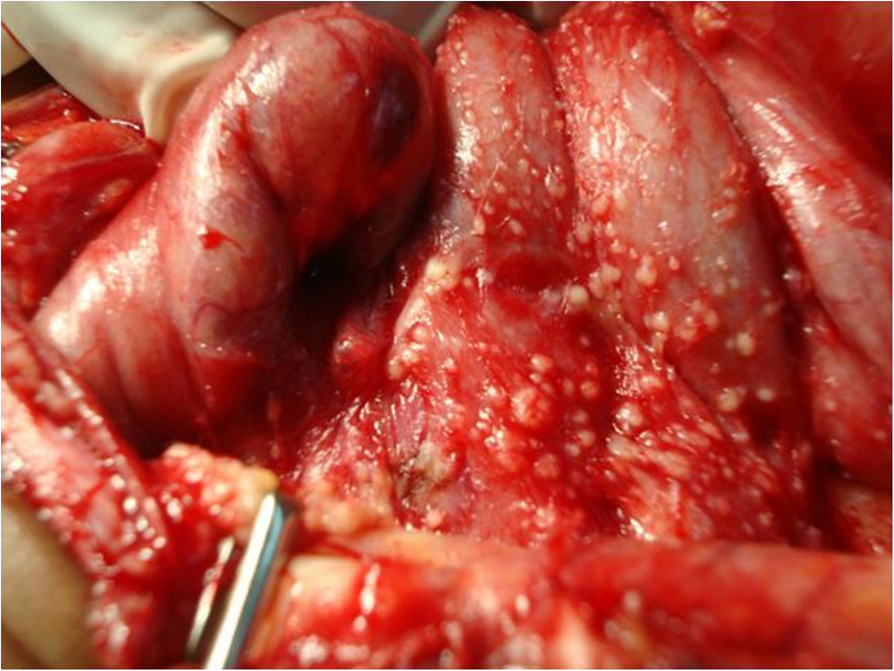
**Intraoperative findings.** Miliary seedlings on peritoneum and serosal surface of bowel with dense adhesions.

At laparotomy the peritoneal cavity was difficult to enter with thick adhesions and miliary seedlings (Figure 5). Frozen section facilities were not available however biopsies were taken for tissue diagnosis, leaving the uterus and ovaries intact. Histology revealed caseating granulomas with epithelioid and Langhan’s type giant cells. The Ziehl–Neelsen stain for AFB was negative. PCR testing was unavailable however the patient’s symptoms resolved and the CA125 levels normalized after 2 months of antituberculosis therapy. She is to continue her treatment for a total of 6 months.

## Conclusions

Signs and symptoms typically associated with advanced ovarian carcinoma include abdominal distension, ascites and pelvic or adnexal masses [8]. Many of these women go on to have radical surgery due to the difficulty of definitive preoperative diagnosis of ovarian cancer and the low negative predictive value of ascitic fluid cytology [6]. Abdominal tuberculosis can present with a similar clinical scenario, with most cases diagnosed incidentally at laparotomy [9]. This has often led to unnecessary extensive surgery, frequently in women of reproductive age [6,7].

CA125 lacks specificity, elevated in many conditions; including tuberculosis [10]. One study showed that CA125 titres higher than 1,000 U/ml correlated with malignancy [11] however there was a reported case of peritoneal tuberculosis with a CA125 level of 1,081 U/ml [12]. This means that absolute CA125 levels are not definitive in determining malignant versus non malignant causes. Although CA125 levels are useful in monitoring response to therapy in ovarian carcinoma [13], there has been no report of CA125 levels declining without treatment in malignant conditions.

Chest radiographs can be normal in patients with peritoneal tuberculosis, approximately 40% of the time [14]. Abdominal CT in tuberculous peritonitis typically shows smooth, strongly enhancing peritoneal thickening and a dirty omentum. Peritoneal carcinomatosis however commonly shows nodular peritoneal thickening and a nodular or caked omentum. Other findings that suggest a diagnosis of tuberculosis include dense ascites, caseous nodes and soft-tissue mesenteric and omental infiltration [15].

There are high false negative rates for tuberculosis skin tests and AFB detection in pleural and peritoneal fluid [9,16]. For preoperative detection of tuberculosis, ascitic fluid adenosine deaminase (ADA) [17] and PCR analyses have proven to be useful [16], however these tests may not be available in all settings. Ultrasound guided tru-cut biopsy has also been shown to be a valuable first line approach [3]. Diagnostic laparoscopy or laparotomy is usually necessary however for definitive diagnosis, where intraoperative frozen sections [12,16] can aid in avoiding unnecessary extensive surgery.

In conclusion, abdominal tuberculosis should be considered in the differential diagnosis of adnexal masses, ascites or elevated CA125, even with negative bacteriologic and cytologic studies. Particular radiological findings can help differentiate between tuberculosis and ovarian malignancy. Ascitic fluid ADA levels and PCR can also aid in the diagnosis. If these tests are negative and clinical suspicion remains, laparoscopy or laparotomy using frozen sections can confirm the diagnosis. Major unnecessary surgery can therefore be avoided using minimally invasive methods preoperatively or frozen sections intraoperatively. Clinicians must consider this important differential diagnosis of tuberculosis, particularly in endemic areas and immigrant populations, as it can closely mimic ovarian carcinoma.

## Consent

Written informed consent was obtained from the patient for publication of this case report and accompanying images. A copy of the written consent is available for review by the Editor of this journal.

## Abbreviations

CT: Computed tomography; PCR: Polymerase chain reaction; ADA: Adenosine deaminase.

## Competing interests

The authors declare that they have no competing interests.

## Authors’ contributions

GN, DN, NB and PS were clinicians in the management of the patient. MG was the radiologist who evaluated the imaging studies. All authors read and approved the final manuscript.
